# The effect of bubble nucleation on the performance of a wickless heat pipe in microgravity

**DOI:** 10.1038/s41526-022-00197-5

**Published:** 2022-04-28

**Authors:** Jiaheng Yu, Anisha Pawar, Joel L. Plawsky, David F. Chao

**Affiliations:** 1 The Howard P. Isermann Department of Chemical and Biological Engineering, Rensselaer Polytechnic Institute, Troy, NY 12180 USA; 2 NASA Glenn Research Center, Cleveland, OH 44135 USA

**Keywords:** Mechanical engineering, Chemical engineering

## Abstract

Bubble nucleation was investigated in a 20-mm-long, wickless heat pipe on the International Space Station. Over 20 h of running experiments using pentane as the working fluid, more than 100 nucleation events were observed. Bubble nucleation at the heater end temporarily boosted peak pressures and vapor temperatures in the device. At the moment of nucleation, the heater wall temperature significantly decreased due to increased evaporation and the original vapor bubble collapsed due to increased pressure. A thermal model was developed and using the measured temperatures and pressures, heat transfer coefficients near the heater end of the system were extracted. Peak heat transfer coefficients during the nucleation event were over a factor of three higher than at steady-state. The heat transfer coefficient data were all collapsed in the form of a single, linear correlation relating the Nusselt number to the Ohnesorge number.

## Introduction

A heat pipe is an efficient heat transfer device that operates by recirculation of a fluid through liquid-vapor phase change and capillary forces. Heat pipes have found extensive application in space research where high heat flux rates are required for cooling over long distances. With no moving parts and a low need for maintenance, heat pipes are an attractive alternative to traditional heat exchangers^1–5^.

The Constrained Vapor Bubble (CVB) experiment studied the heat transfer behavior of a transparent, wickless heat pipe aboard the International Space Station to understand the influence of interfacial and intermolecular forces on the vapor-liquid distribution in the absence of gravity^6,7^. The CVB consisted of a square fused silica cuvette, so the interface between the vapor and liquid inside the heat pipe could be observed and the liquid film thickness on the walls of the device measured using interferometry. The high temperature at the heater end caused the working fluid to evaporate, and the vapor condensed at the cold end, which was kept at a temperature well below the saturation temperature of the vapor. Capillary forces arising from the sharp corners of the cuvette returned the liquid back to the hot end^8–11^. In one incarnation of the experiment, bubble nucleation, akin to nucleate boiling, was observed and is the subject of this paper.

Boiling is a ubiquitous phase change phenomenon occurring in superheated fluids^12–19^. Many researchers have studied the growth and departure of bubbles on a heated surface under various temperature and pressure conditions to understand the process of nucleate boiling in detail^20–23^. Most experiments have been conducted under constant pressure conditions in Earth’s gravity, though over the years, there have been quite a few experiments in low gravity and microgravity conditions^20,24^. Numerical models were also developed to predict and evaluate the heat transfer mechanisms during nucleate boiling^25–27^. The curvature of the meniscus underneath a bubble, the effect of interfacial forces, the thermal resistance at the liquid-vapor interface, the role of Marangoni convection^28^, and the turbulence induced by the bubble growth are all found to be the related factors that affect the heat transfer behavior of boiling systems.

Rapid heating of a liquid leads to instabilities which can be observed in the form of abrupt boiling^29–32^. Orrit et al. performed experiments on a single 80 nm gold nanoparticle to investigate the boiling regime at a nanoscale level^33^. They observed how the behavior of a nanobubble changes on varying the heater power and found that 120 µW is the boiling threshold above which vapor nanobubbles form and disappear. Monde et al. applied a theoretical model to study the homogenous nucleate boiling regime in ethanol at atmospheric pressure^34^. They calculated the time to boiling explosion and the liquid temperature inside a liquid control volume and found their results were in close agreement with the literature. Hasan et al.^35^ observed explosive nucleation of bubbles in microgravity as part of NASA’s Zero Boil-Off Tank experiment. They reported that at relatively low heat fluxes, high values of superheat could be sustained punctuated periodically by rapid nucleation and growth of a vapor bubble. Reza and Zhang carried out molecular dynamics simulations of argon on a copper surface^36^. The aim was to study the effect of nanostructures on heat transfer. They found that the nanostructured surface provides an advantage in heat transfer over a flat surface due to the increased interaction between the liquid and the structured surface. The temperature at which explosive boiling occurs was found to depend on the size of the nanostructures.

A rapid drop in the surface temperature was observed in many nucleate boiling experiments^37,38^. A significant amount of heat was absorbed during evaporation creating a bubble in a short time period. Meanwhile, the bubble formation process agitated the flow in the liquid phase, which also enhanced the heat transfer process. However, in wick-structured heat pipe systems, nucleate boiling is generally considered to be a detrimental factor for heat pipe performance^39^. At high heat fluxes, bubbles generated by nucleate boiling occur within the wick and block the cooled liquid from returning to the heated end. This circumstance is referred to as the boiling limit of the heat pipe, which restricts the maximum operating temperature^40^. In contrast, experiments using wickless heat pipes/thermosyphons showed an increase in the heat flux was achieved due to boiling in the bulk liquid phase^41,42^.

This paper extends the previous research on nucleate boiling in the CVB system in microgravity to seven experimental “bins” covering the range of nucleation events that occurred. The work emphasizes the analysis of the effect of nucleation on heat pipe performance^43^. A one-dimensional heat transfer analytical model was developed based on the work by Bowman and Maynes, which allowed us to evaluate an average heat transfer coefficient in the evaporation region and to compare that with the strength of the nucleation event^44^. We found nucleation reduced the heater wall temperature and boosted the heat transfer coefficient significantly though temporarily. Stronger nucleation events in the CVB system led to higher heat transfer coefficients and more effective heat pipe performance. Meanwhile, stronger nucleation also minimized the difference between the heater wall temperature and saturation vapor temperature. Box 1 presents the nomenclature table for all variables used in this article.

Box 1 Nomenclature

**Table:** 

Roman symbols	
*A*_*c*_	cross sectional area of the cuvette wall (m^2^)
*h*_*in*_	average internal heat transfer coefficient (W/m^2^ K)
*k*	thermal conductivity (W/m K)
*L*	characteristic length (m)
*Nu*	Nusselt number
*n*_*p*_	the number of pressure data in a nucleation cycle
*n*_*T*_	the number of temperature data in a nucleation cycle
*Oh*	Ohnesorge number
*P*	system pressure (Pa)
*P*_*in*_	inside perimeter of the cuvette (m)
*P*_*initial*_	initial stage system pressure of the nucleation cycles (Pa)
*P*_*out*_	outside perimeter of the cuvette (m)
*q*_*cond*_	conduction heat transfer rate (W)
*q*_*out,rad*_	thermal radiation heat transfer rate (W)
*q*_*in*_	internal heat transfer rate (W)
*q’*_*cond*_	conduction heat flow per unit length (W/m)
*q’*_*out,rad*_	outside radiation heat flow per unit length (W/m)
*q’*_*in*_	internal heat transfer flow per unit length (W/m)
*s*_*p*_	sampling rate of the pressure data (s^−1^)
*s*_*T*_	sampling rate of the temperature data (s^−1^)
*T*	temperature (K)
*t*	time (s)
*TC*#*n*	the n^th^ thermocouple
*T*_*v*_	saturation vapor temperature (K)
*T*_*∞*_	temperature of the external environment (K)
*∆T*	superheat (K)
*x*	distance (m)
Greek symbols	
*α*	thermal diffusivity of the liquid (m^2^/s)
*ε*	emissivity of the cuvette material
*μ*	dynamic viscosity of the liquid (N s/m^2^)
*ρ*	density (kg/m^3^)
*σ*	Stefan–Boltzmann constant (W/m^2^ K^4^)
*σ*_*l*_	surface tension of the liquid (N/m)

## Methods

### Hardware configuration and sampling rate settings

**Fig. 1:**
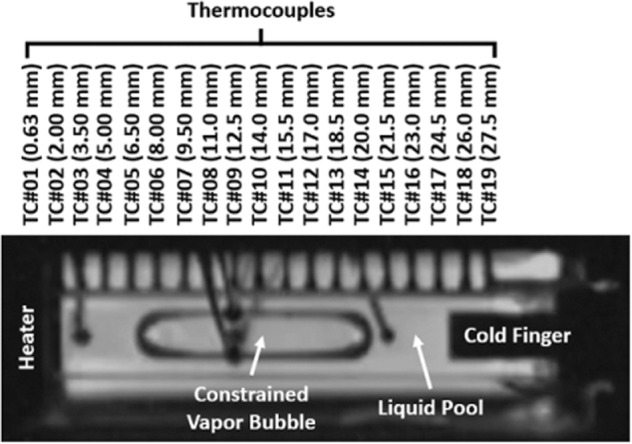
Surveillance Image of the CVB system. A snapshot by the surveillance camera of the partially filled 20 mm CVB module at the steady state. 19 thermocouples, TC#01 to TC#19, were installed on the sidewall of the cuvette. The spacing of the thermocouples is 1.5 mm except for the first thermocouple, TC#01, which was embedded in the wall between the heater and the working liquid.

In the CVB experiment, a fused silica cuvette was used as a transparent wickless heat pipe. The cuvette had a cross-sectional dimension of 5.5 × 5.5 mm outside and 3.0 × 3.0 mm inside the cavity space. The working fluid of the heat pipe was pure pentane. The constrained bubble in the cuvette section was generated by evacuating the system and then partially filling the liquid into the CVB module. Videos and images of the experiments were recorded by a wide-angle surveillance camera to observe the migration of the bubble inside the cuvette. The temperature profile along the main axis of the heat pipe was measured by 19 thermocouples installed on one side of the cuvette (Fig. 1). Fig. S.4 shows the wells that were drilled on the cuvette surface. The beads of the type-E thermocouple junctions were embedded in the corresponding wells to ensure good contact for temperature measurements. The temperature of the heater wall was measured by the first thermocouple, TC#01, at 0.63 mm. The last thermocouple, TC#19 at 27.5 mm, detected and recorded the temperature of the cold finger. From TC#01 to TC#19, the effective length of the cuvette for the investigation is bounded in this region. The sampling rate of the temperature readings was 0.5 s^−1^ ≤ *s*_*T*_ ≤ 1.0 s^−1^, and the accuracy of the thermocouples was less than ±0.5 °C. During the entire nucleation experiment (00:00:00–20:21:17 Oct 17, 2010), the power input to the heater was set at 1.535 W and this maintained the steady-state temperature of the heater wall at ~117 °C. Meanwhile, the temperature at the cold end was controlled by the cold finger and was held at 20 ± 0.1 °C. The internal pressure of the CVB system was measured by a pressure transducer that was attached downstream of the cuvette section. The specified accuracy of the sensor is ± 0.69 kPa. The sampling rate of the system pressure readings is *s*_*P*_ = 1.0 s^−1^. The experiment was conducted onboard the International Space Station in the Fluids Integrated Rack (FIR) to minimize the influence of gravitational force and focus on analyzing the effect of interfacial forces and nucleate boiling. The Light Microscopy Module in the FIR was used for the optical observations. A more detailed description of the apparatus and operation of the explosive nucleation CVB experiment can be found in the previous publication^43^.

## Results

### Overview of the microgravity CVB experiment

The CVB experiment was performed aboard the International Space Station to understand how microgravity affects the performance of a self-contained, evaporating and condensing system, like a heat pipe. The microgravity conditions minimized the effects of body forces on the system and allowed interfacial forces to dominate the behavior. The gravitational acceleration was ~0.19 µg over the experimental period and so the Bond number was effectively zero. More information on the microgravity environment can be found in references^43,45,46^. In the CVB experiments, several different lengths of heat pipe were explored. Following bubble disruption during launch, getting all the vapor back into a single bubble and coaxing that bubble to adhere to the heater wall involved first turning on the condenser to cool the back end of the system and condense any vapor that remained there, and then turning on the heater to force the bubble to migrate toward it. This procedure worked for longer versions of the heat pipe but failed in the shortest, 20 mm-long, version due to strong interfacial forces that created flows in the corners of the device and prevented the bubble from attaching to the heater wall^8–11,45–50^. Instead, bubbles would randomly nucleate at the heater wall, merge with the original vapor bubble, and eventually the new, single bubble would migrate back to the center of the device. In a previous paper, we analyzed a single such event^43^. In this paper, we were interested in analyzing many more and using more recently developed thermal analyses to quantify how the internal heat flow and heat transfer coefficient changed during the course of these events.

Figure 1 shows the apparatus used in the experiment. The 20 mm module, made of fused silica, was evacuated and then partially filled with pure liquid pentane as the working fluid. Power inputs to the heater (1.535 W) and the temperature of the condenser (19 °C) were held constant. The power input was chosen as the best compromise between the frequency of nucleation events and not exceeding the pressure limits that would automatically shut down the experiment. Temperature profiles along the main axis of the heat pipe were measured by 19 thermocouples attached within one sidewall, the temperature of the external environment was also recorded, and the system pressure was monitored and recorded by a pressure sensor located downstream of the condenser end. Video of a number of these events were obtained using a surveillance camera and can be found in the Supplementary Information section. The experimental system was not designed to study nucleation and so pressure, temperature, and video were not fast enough to record all the details of the nucleation event. Still, sufficient measurements were made throughout each event to provide some level of understanding.

### Bubble nucleation in the CVB system

**Fig. 2:**
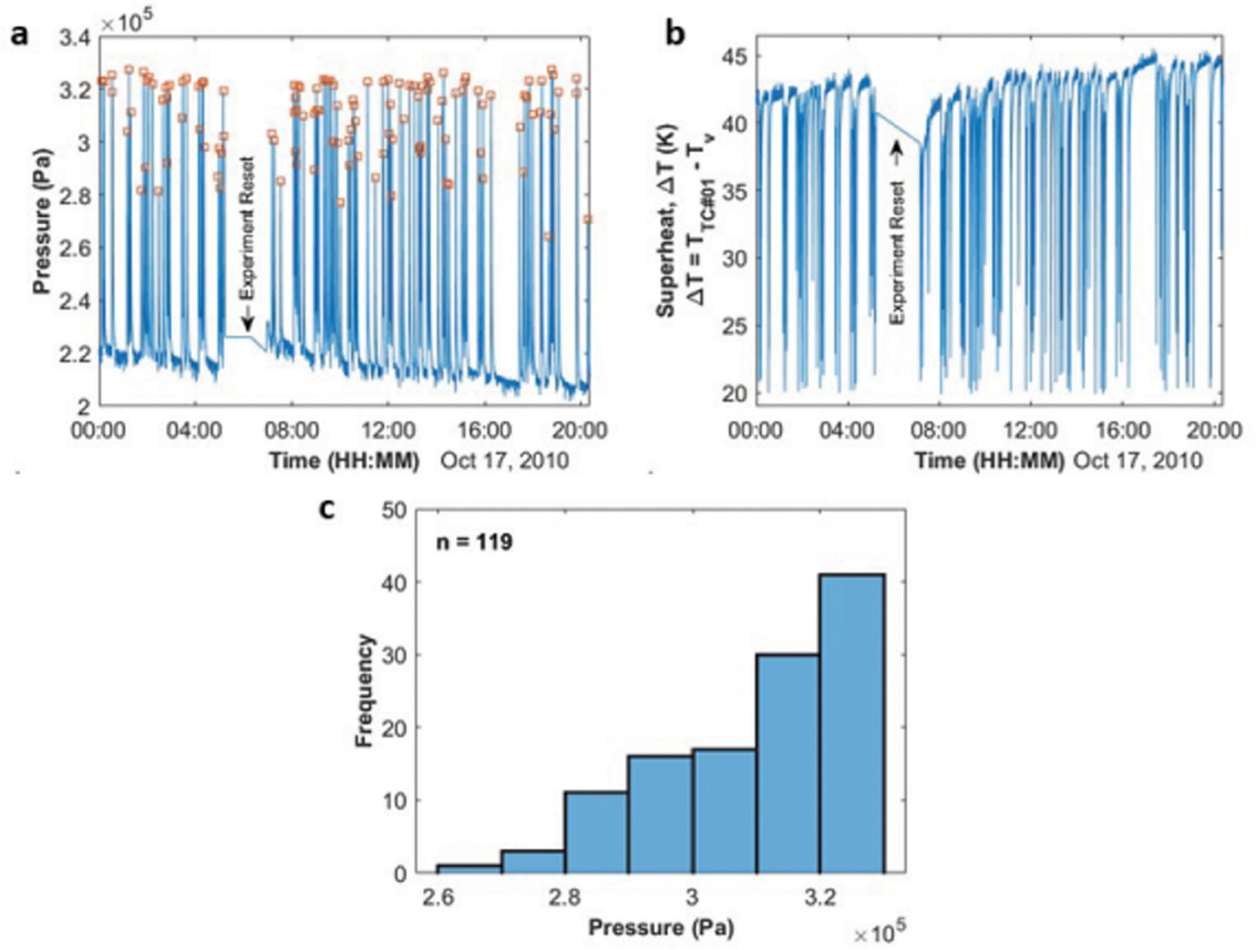
Pressure data from the nucleation CVB experiment. **a** Pressure profile across the entire experiment duration (00:00:00–20:21:17 Oct 17, 2010). Peak pressure in each cycle is labeled using square dots. **b** Histogram of all the 119 cycles observed during the entire experiment. With a bin size of 0.1 × 10^5 ^Pa, there are 7 bins in the diagram. The 7 intervals are (2.6 × 10^5 ^Pa–2.7 × 10^5 ^Pa), (2.7 × 10^5 ^Pa–2.8 × 10^5 ^Pa), (2.8 × 10^5 ^Pa–2.9 × 10^5 ^Pa), (2.9 × 10^5 ^Pa–3.0 × 10^5 ^Pa), (3.0 × 10^5 ^Pa–3.1 × 10^5 ^Pa), (3.1 × 10^5 ^Pa–3.2 × 10^5 ^Pa), and (3.2 × 10^5 ^Pa–3.3 × 10^5 ^Pa). **c** Superheat (∆*T*) during the nucleation events.

Figure 2a shows the pressure profile across 20 h of the nucleation experiment. The square dots represent the times associated with the local peak pressures, in each nucleation cycle. These peak pressures were not the pressure at the actual nucleation event itself but the point where the newly nucleated bubble and original bubble merged. The nucleation event and growth of the new bubble were too fast for us to record. 119 cycles were observed over the course of 20 h. In Fig. 2b, the data shows that peak pressures ranged from 2.6 × 10^5 ^Pa to 3.3 × 10^5 ^Pa which we now bin into seven wells separated by 0.1 × 10^5 ^Pa. 60% of the cycles exhibited peak pressures between 3.1–3.3 × 10^5 ^Pa with only four cycles between 2.6–2.8 × 10^5 ^Pa. Figure 2c shows the superheat, ∆*T*, associated with each of these scenarios. The superheat was defined as the temperature difference between the heater wall, *T*_*TC*#01_, and the saturation temperature, *T*_*v*_, associated with the measured pressure. This latter temperature was determined using the Antoine equation for pentane. Notice that the superheat increased between each nucleation event. The experiment was eventually stopped to insure we could revisit the module and attempt to run it later as a heat pipe.

**Fig. 3:**
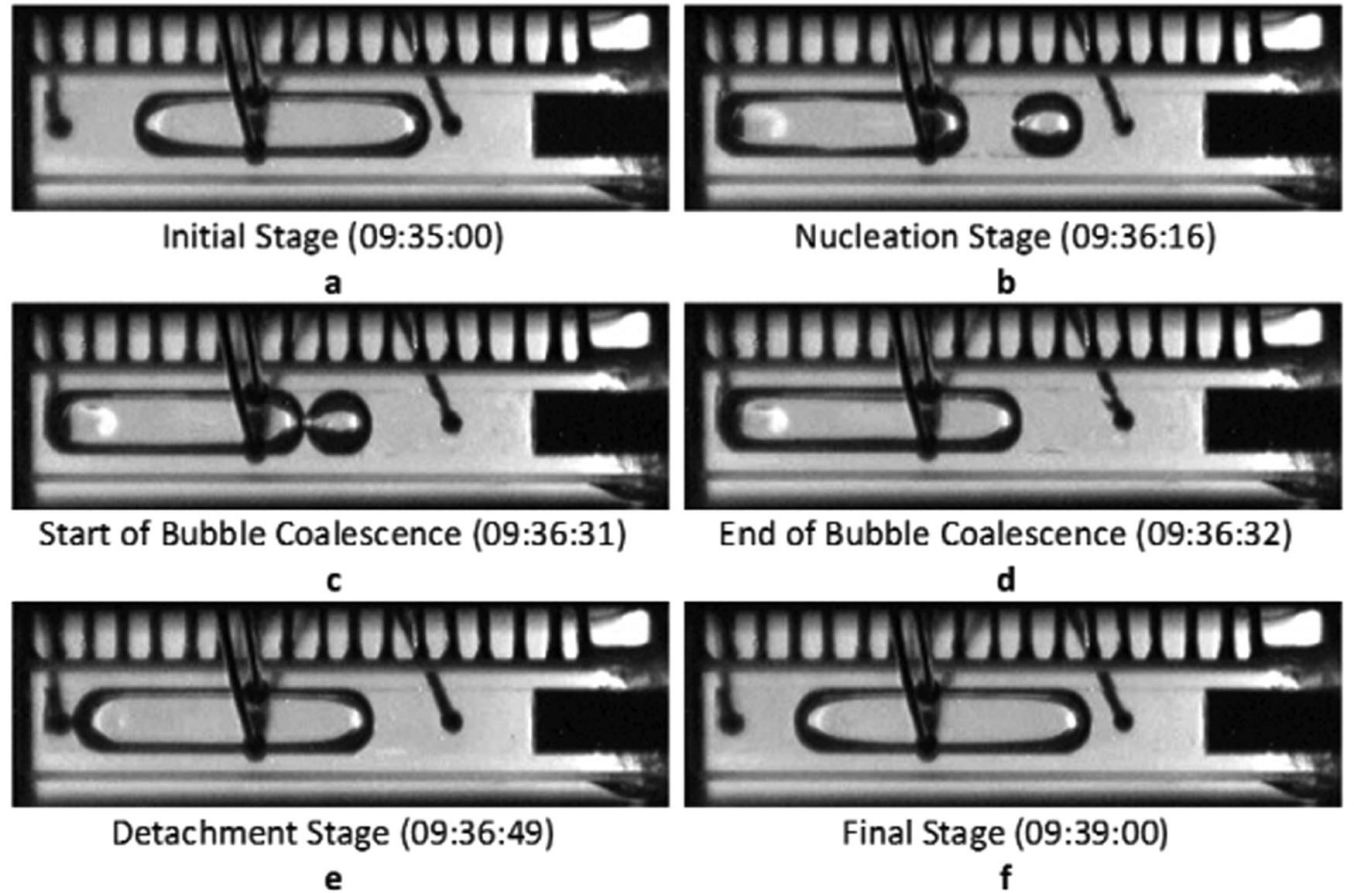
Nucleation event milestones^43^. **a** Initial state with bubble near the middle of the device. **b** Moment of the nucleation event. A new bubble was generated at the hot end by evaporation of the liquid phase. The original bubble in (**a**) collapsed with the sudden increase in the system pressure. **c** The start of the bubble merging process for the new and the original bubble. **d** The end of the merging process for the two bubbles. At this moment, the combined bubble was still attached to the heater wall. **e** Progression of the bubble back toward its initial location. **f** Return of the bubble to its initial location.

**Fig. 4:**
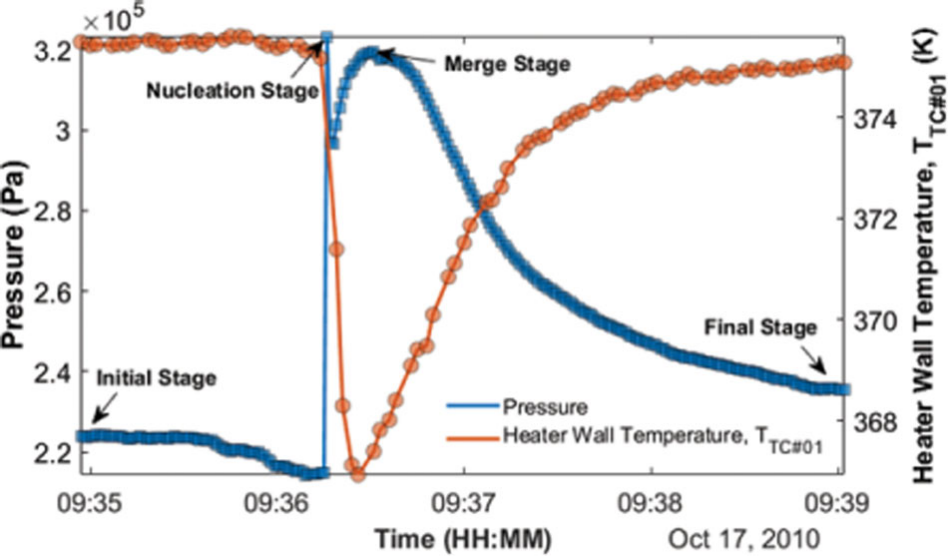
Pressure and temperature profiles for the nucleation event shown in Fig. 3. Profiles of the system pressure and the heater wall temperature for the nucleation cycle (09:34:57–09:39:02) illustrated in Fig. 3.

The details of a single nucleation event were discussed in detail in a previous publication^43^, so only a brief review is provided here. Figure 3 consists of images illustrating the key features of a typical nucleation cycle, and Fig. 4 depicts the changes in pressure and heater wall temperature that occurred during this nucleation event. The low thermal conductivity of the glass walls coupled with radiation losses from the cell to the surroundings established a large temperature gradient between the hot and cold ends. Strong capillary return flow prevented the vapor bubble from attaching to the heater wall (Fig. 3a). The flow at the tail of the bubble is best observed in the single nucleation event video in Supplementary Information. The superheated liquid at the heater wall would occasionally promote the nucleation of a vapor bubble there as shown in Fig. 3b. The pressure increase associated with the nucleation of the new bubble collapsed the original bubble so that we observed two bubbles simultaneously inside the cuvette (Fig. 3b). In Fig. 4, the new bubble near the heater wall significantly boosted the internal pressure due to strong evaporation. At the same time, it dramatically cooled down the heater wall temperature. The vapor in the original bubble condensed to accommodate the sudden increase in pressure due to nucleation. The decrease in the pressure profile (Fig. 4) right after nucleation corresponded to a relaxation following the collapse of the original bubble. The remnant of the original bubble then moved towards the new bubble, and the two bubbles merged into one (Fig. 3c, d). At the stage of Fig. 3d, the CVB system behaved similarly to a conventional heat pipe but then the bubble detached from the heater wall (Fig. 3e) and the pressure began to decrease due to a decrease in the rate of evaporation as the bubble moved toward the condenser end. The curvature of the corner liquid on the left-hand side of the bubble is higher than that on the right in Fig. 3d and this established a capillary flow that brought liquid from the condenser end. This replenished liquid to the hot end and helped separate the bubble from the heater wall. The bubble migrated back to its starting position (Fig. 3f). Thus, Fig. 3f was the final state of one nucleation cycle and the initial state of the next nucleation cycle. The process is most easily seen in the videos that can be found in the Supplementary Information.

We selected 20 out of 119 cycles representing every bin in Fig. 2b. We only picked those events where there were clear initial and final stages though in some instances, nucleation events, appeared in bursts (see Supplementary Information for a video). Fig. S.1 highlights the 20 cycles we selected for the analysis, and Table S.1 summarizes the basic information of each cycle. Since there are only 7 peak pressure groups in the histogram of Fig. 2b, we again picked 7 representative cycles to show in the figures.

The duration of the nucleation phenomenon in the experiment was much faster than any of the experiment’s instruments could detect. The sampling rate for the pressure readings (≈1 s^−1^) and temperature readings (≈0.5 s^−1^) was not high enough to catch the moments of nucleation for all events and nucleation and bubble merging in the video occurred within one video frame. It would be difficult to identify, even in Fig. 4, if the 3.23 × 10^5 ^Pa first peak was the real internal pressure at nucleation. This pressure was very close to the safety cutoff of the system and luckily occurred too fast for the software to register. Outside of the nucleation event, the rest of the cycle was slow enough to record and so we characterize the events using the second peak pressure which occurs when the two bubbles coalesce.

**Fig. 5:**
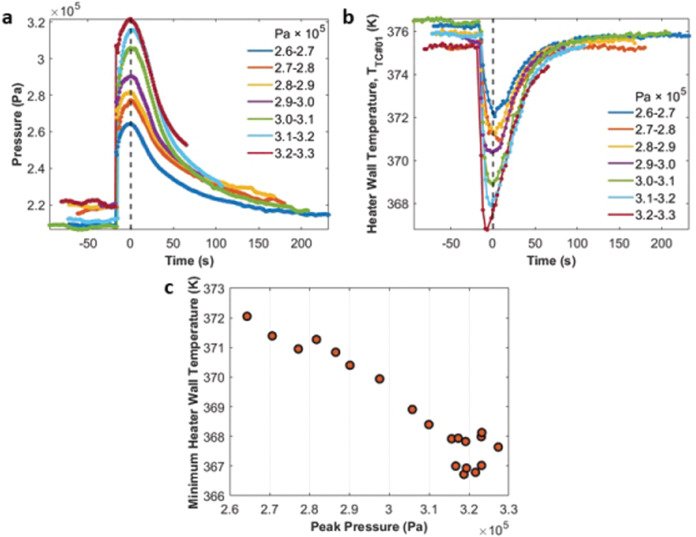
System pressures and heater wall temperatures. **a** Pressure profiles for the 7 peak pressure groups at the sampling times of the pressure transducer. The time at the peak pressure is used to align the profiles. **b** Change of the heater wall temperatures with time for the 7 peak pressure groups. **c** The minimum heater wall temperatures for the 20 cycles in the 7 peak pressure groups.

Figure 5a, b align the 7 pressure profiles and the heater wall temperature profiles at the peak pressure times (dashed line). From the different pressures at the coalescence stage, we can infer that nucleation boosted the system pressure to different levels driven by an increase in the amount of vapor generated. As mentioned earlier, the temperature of the heater wall decreased significantly during the nucleation event because a new bubble was generated at the heater end. It is important to notice that the minimum heater wall temperature in each group generally precedes the peak pressure at bubble coalescence. This indicates that this peak pressure was due not only to the initial nucleation event but also due to evaporation that occurred while the new, single bubble was attached to the heater wall. The lower minimum value of the heater wall temperature is found in the higher peak pressure group. In Fig. 5c, the summary of all the 20 cycles shows that the decrease in the minimum heater wall temperature due to more severe nucleation events (higher peak pressures) prevailed throughout the entire experiment. To help quantify this observation, we use a one-dimensional (1-D) heat transfer model to determine the heat flow rates to and from the internal walls of the device and to calculate a heat transfer coefficient at the evaporator throughout each nucleation event.

## Discussion

**Fig. 6:**
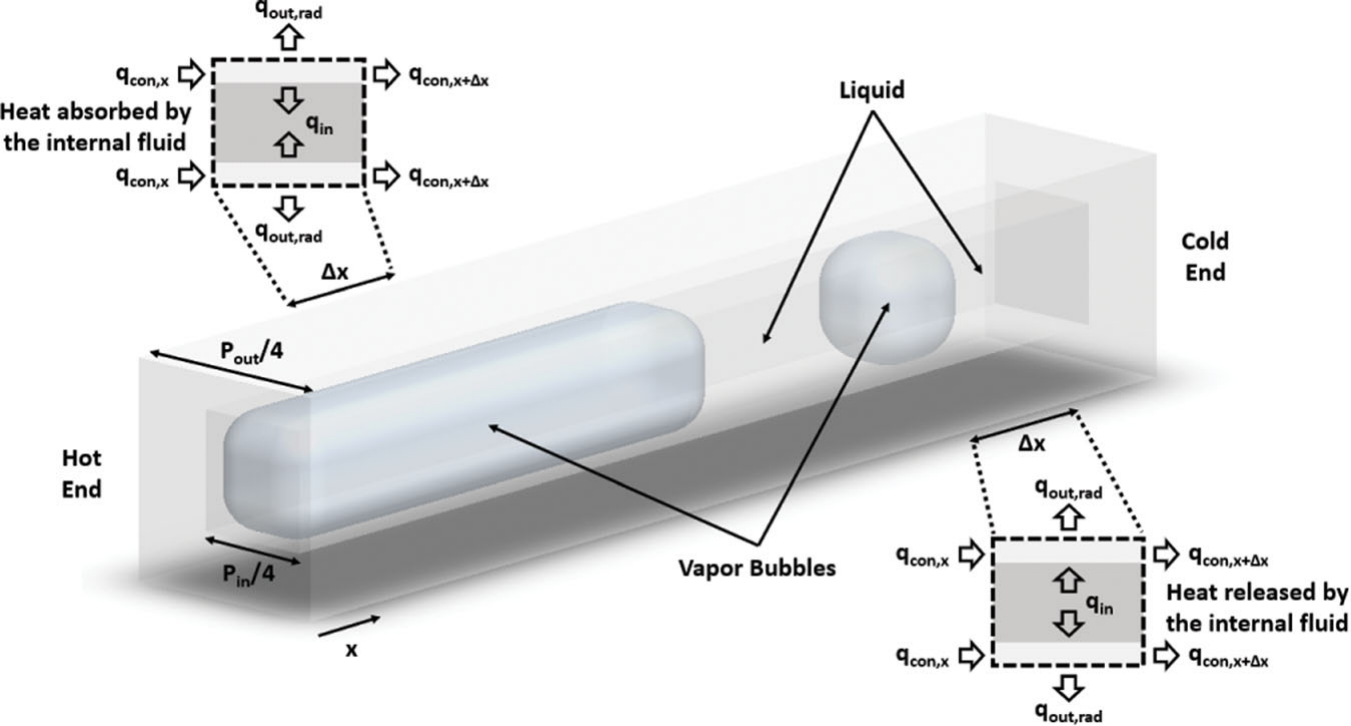
One dimensional heat transfer model geometry. Schematic of the CVB heat pipe with various heat transfer mechanisms. Heat was absorbed by the internal fluid near the hot end but was released near the cold end.

The basic structure of the thermal model is similar to a standard fin with the addition of convection and phase change inside the heat pipe. The model is inspired by the one originally developed by Bowman and Maynes^44^. Natural convection outside the cuvette is neglected due to the microgravity environment. Therefore, three heat transfer mechanisms are included as shown in Fig. 6: conduction of heat through the glass wall from the hot end to the cold end (*q*_*cond*_); thermal radiation from the external surface to the surrounding environment (*q*_*out,rad*_); phase change and convective flow of the working fluid (*q*_*in*_) internally.

A differential equation presented in Eq. (1) was derived based on the control volume in Fig. 6.

1 $$\underbrace {kA_c\frac{{d^2T}}{{dx^2}}}_{q_{cond}^\prime } - \underbrace {P_{in}h_{in}\left( {T - T_v} \right)}_{q_{in}^\prime } - \underbrace {\sigma \varepsilon P_{out}\left( {T^4 - T_\infty ^4} \right)}_{q_{out,rad}^\prime } = 0$$

In Eq. (1), *T*_*v*_ represents the saturation vapor temperature, and *h*_*in*_ is an average heat transfer coefficient that is used to evaluate the performance of the heat pipe, primarily in the region near the heater end. Although *T*_*v*_ changed with time as the system pressured varied, it is assumed to be relatively constant within the heat pipe at any moment. The saturation vapor temperature was estimated from the pressure profile using the Antoine equation, Eq. (2), when changes to the operating parameters of the CVB system are changing relatively slowly. The parameters for the Antoine equation are listed in Table S.2 in the Supplementary Information^49,50^.

2 $$T_v = \frac{B}{{A - log_{10}P}} - C$$

**Fig. 7:**
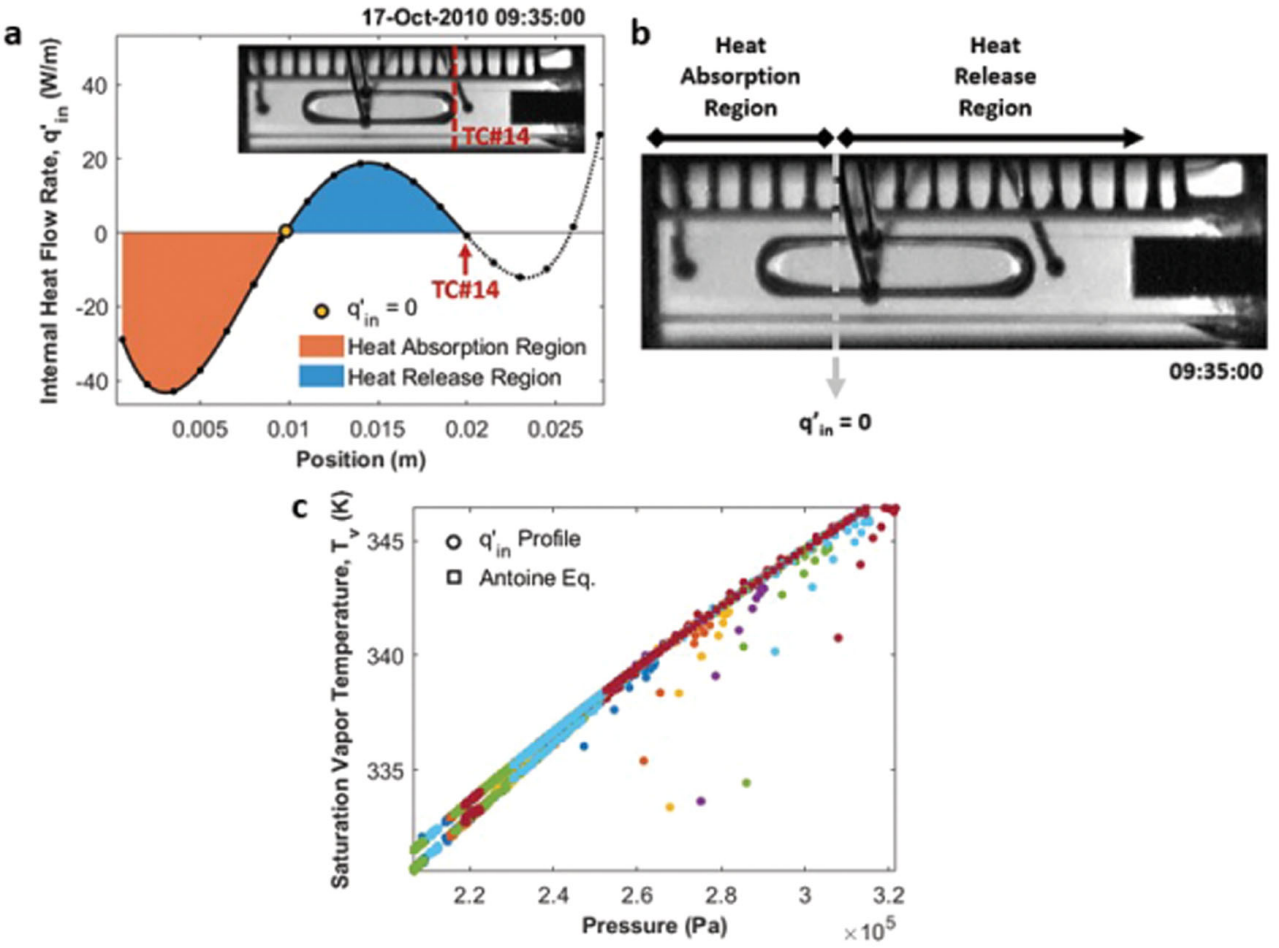
*q’*_*in*_ profile and saturation vapor temperature. Two regions with different heat transfer behaviors are identified in the CVB module based on the position where *q’*_*in*_ = 0. **a** Internal heat transfer profile, *q’*_*in*_, at the initial stage of the nucleation cycle shown in Fig. 3a. **b** Surveillance image showing the physical location where the first *q’*_*in*_ = 0 occurs and identifying the absorption and release regions. **c** Saturation vapor temperature calculated using the *q’*_*in*_ profile and the Antoine equation for the 7 peak pressure groups.

A second method was also used to obtain a value for *T*_*v*_ from the *q’*_*in*_ profile. By rearranging Eq. (1), we can calculate *q’*_*in*_ from the other two heat transfer components as shown in Eq. (3). Figure 7a demonstrates the *q’*_*in*_ profile at the time of Fig. 3b. The region of the heat pipe where *q’*_*in*_ < 0 means heat was gained by the internal fluid from the glass wall and the region where *q’*_*in*_ > 0 means heat was lost by the internal fluid to the glass wall. Thus, the heat pipe, such as it is, can be segmented into two primary sections: a heat absorption region and a heat release region, as illustrated in Fig. 7b. The second point where *q’*_*in*_ = 0 corresponds to the end of the vapor bubble. The transition point between the two, colored sections, where *q’*_*in*_ = 0, provides a value for *T*_*v*_ within the vapor bubble and we use the distance between the location of *T*_*v*_ and the heater wall as the characteristic length of our device. Figure 7c compares the calculation of *T*_*v*_ from the two methods for the 7 groups. The sampling rate of the temperature data was slower than that of the pressure data and so saturation vapor temperatures estimated from the *q’*_*in*_ profiles may not exactly mirror the rapid changes in pressure that occur at the time of nucleation or bubble coalescence. Thus, using the *q’*_*in*_ profiles, the measurements during the time interval between the initial stage and nucleation stage deviate from the bulk data, and these points are located at the higher-pressure regions in the cycle. However, the majority of the data points where changes are relatively slow are highly matched with the saturation vapor temperatures calculated by the Antoine equation, so the thermal model can still predict and describe the dynamic changes in the CVB system over most of the event.

3 $$q_{in}^\prime = q_{cond}^\prime + q_{out,rad}^\prime$$

**Fig. 8:**
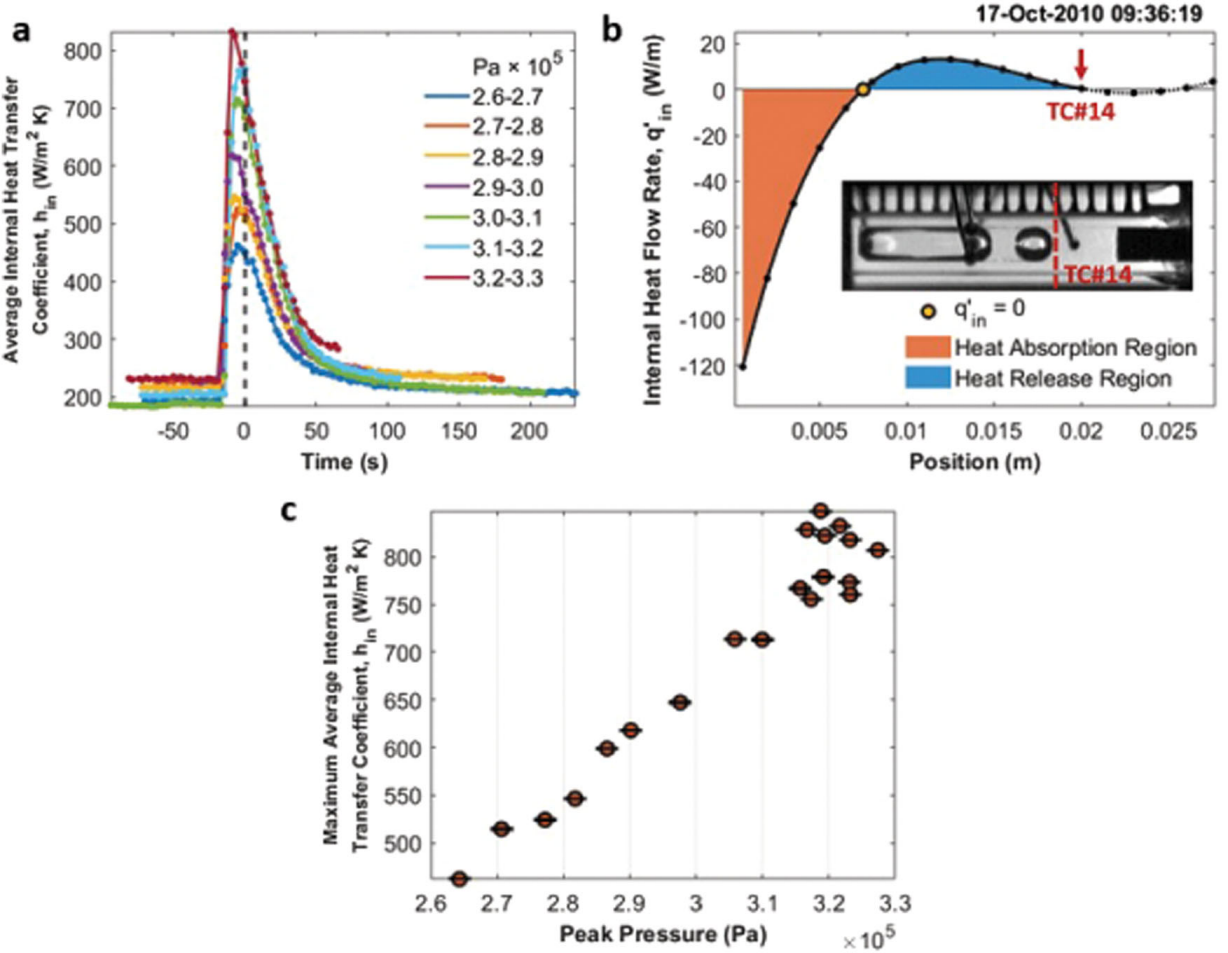
Average internal heat transfer coefficient, *h*_*in*_, and *q’*_*in*_ profile at the nucleation stage. **a** Change of the average internal heat transfer coefficient with time in the heat absorption region for the 7 peak pressure groups. The maximum error of the average heat transfer coefficients for the 7 cycles in (**a**) is 1.34 W/m^2^ K. **b** Internal heat flow rate profile, *q’*_*in*_, at the moment of nucleation, Fig. 3b, in the cycle. **c** The maximum average internal heat transfer coefficients for the entire 20 nucleation events in the analysis. Errors are smaller than 0.58 W/m^2^ K.

With *T*_*v*_ determined, *h*_*in*_ can be calculated from Eq. (1) by an iterative numerical method using *h*_*in*_ as the adjustable parameter to fit the model to the temperature profile. Figure 8a shows these heat transfer coefficients for the 7 groups as a function of time. The average heat transfer coefficients increased significantly at the peak pressure times and decreased rapidly after the new and original bubbles merged. Figure 8b presents the *q’*_*in*_ profile at the peak pressure time corresponding to Fig. 3b. Comparing Fig. 7a and Fig. 8b, the heat absorbed by the internal fluid is much higher at the peak pressure times, which verifies the new bubble generated at the hot end by nucleation removed the heat from the glass wall via evaporation. Meanwhile, in Fig. 8a, the maximum heat transfer coefficient in each group is shown to occur at the time of peak pressure for each group. Figure 8c summarizes the relationship between the maximum heat transfer coefficients and peak pressures for all the 20 cycles analyzed.

It is important to notice that the highest heat transfer coefficient occurs slightly ahead of the peak pressure time in each group because the peak pressure represents the stage of bubble coalescence and not the moment of nucleation which absorbs much more heat. Due to the low sampling rate of the temperature data, there was a lag in time for the maximum heat transfer coefficient, reflecting the heat pipe performance at the nucleation stage in the heat absorption region.

The trend in the average heat transfer coefficient for the 7 groups is related to the temperature gradient in the heat absorption region for the internal fluid. Fig. S.2 shows the full temperature profiles for the 7 groups at the peak pressure times near the heater end. Stronger nucleation resulted in higher peak pressure. Therefore, the saturation vapor temperature was higher at the peak pressure time for stronger nucleation cycles, and a longer heat release region was available for heat removal. Meanwhile, the dramatic evaporation at the hot end significantly cooled down the heater wall temperature. In Fig. S.2, the heater wall temperature was lower in the higher peak pressure group (stronger nucleation cycle). Therefore, nucleation in this version of the CVB heat pipe was beneficial to its performance, as long as the bubble remained attached to the heater wall.

**Fig. 9:**
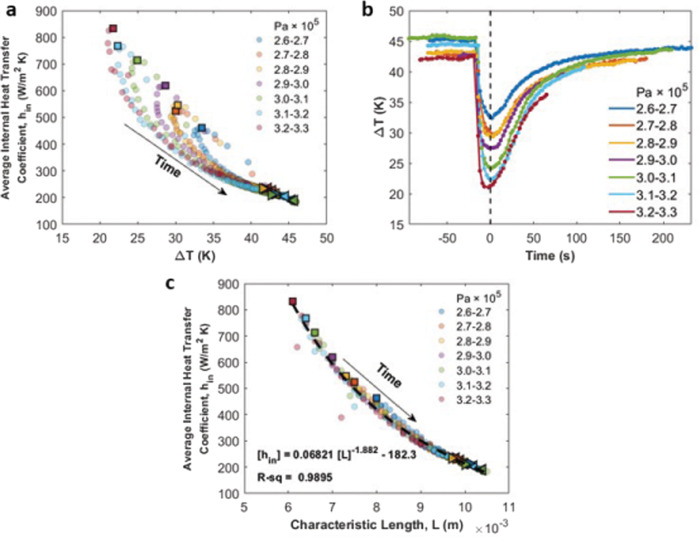
The effect of the temperature gradient and length of the heat absorption region on the average internal heat transfer coefficient. **a** Average internal heat transfer coefficients as a function of temperature gradients in the heat absorption region. The maximum *h*_*in*_ values in 7 group are marked as (▢) representing the nucleation and merge stages (Fig. 3b–d), the *h*_*in*_ values at *t* = −50 s are marked as (◁) representing the initial stage prior to bubble nucleation (Fig. 3a), and the *h*_*in*_ values at *t* = 150 s are marked as (▷) representing the final stage once the bubble returns to the center (Fig. 3f). The three special marker types represent the nucleation and merge stage, initial stage, and final stage of the cycle respectively. **b** Change of the temperature gradient in the heat absorption region with time. **c** Average internal heat transfer coefficients versus the length of the heat absorption region. Special markers share the same definition as in (**a**).

Figure 9a presents the heat transfer coefficients as a function of the superheat in the heat absorption region. The maximum heat transfer coefficient in every group decayed with the superheat but the characteristics of the decay depended on the peak pressure achieved. At the peak pressure times (*t* = 0 s), in Fig. 9b, the difference in ∆*T* for the lowest and highest peak pressure groups is around 11 K. However, at *t* = 150 s, the difference in ∆*T* is around 2 K. Therefore, ∆*T* for the higher peak pressure group decayed faster with time. After the two bubbles merged, both the pressure and the heater wall temperature began returning to their initial values in the cycle. Due to the constant heat input and condenser temperature throughout the entire experiment, the difference in ∆*T* decreased and the tails of all the 7 profiles converged.

The heat transfer coefficient is also related to the length of the heat absorption region, or what we define as the characteristic length for the system. In Fig. 9c, the maximum heat transfer coefficient in each group occurred when the length of the heat absorption region was the shortest. This coincided with the longest heat release region for the internal fluid. Following bubble coalescence, as the system pressure decreased, the saturation vapor temperature decreased, the length of the heat absorption region became longer and so the average heat transfer coefficient decreased with time.

The agreement between *h*_*in*_ and the characteristic length, shown in Fig. 9c, suggested that it might be useful to evaluate the behavior using dimensionless physical quantities. Inspired by the analyses in^51,52^, we found the Ohnesorge number, *Oh*, seems to describe the competition between viscous forces, surface forces and inertial forces driven by the nucleation event (Eq. (4)). In addition, the average heat transfer coefficients and characteristic length can be used to define a Nusselt number (Eq. (5)) in the heat absorption region.

4 $$Oh = \frac{\mu }{{\sqrt {\left( {\rho \sigma _lL} \right)} }}$$

5 $$Nu = \frac{{h_{in}L}}{k}$$

*μ*, *ρ*, *σ*_*l*_, and *k* are the dynamic viscosity, density, surface tension, and thermal conductivity of the liquid. Based on data from NIST, we assume these physical properties of the liquid remained unchanged over the temperature range of the experiment^53^. The values were determined based on the average temperature of the liquid between the heater wall and condenser, 335 K. Table S.3 provides the values used in the calculation of Eqs. (4) and (5).

**Fig. 10:**
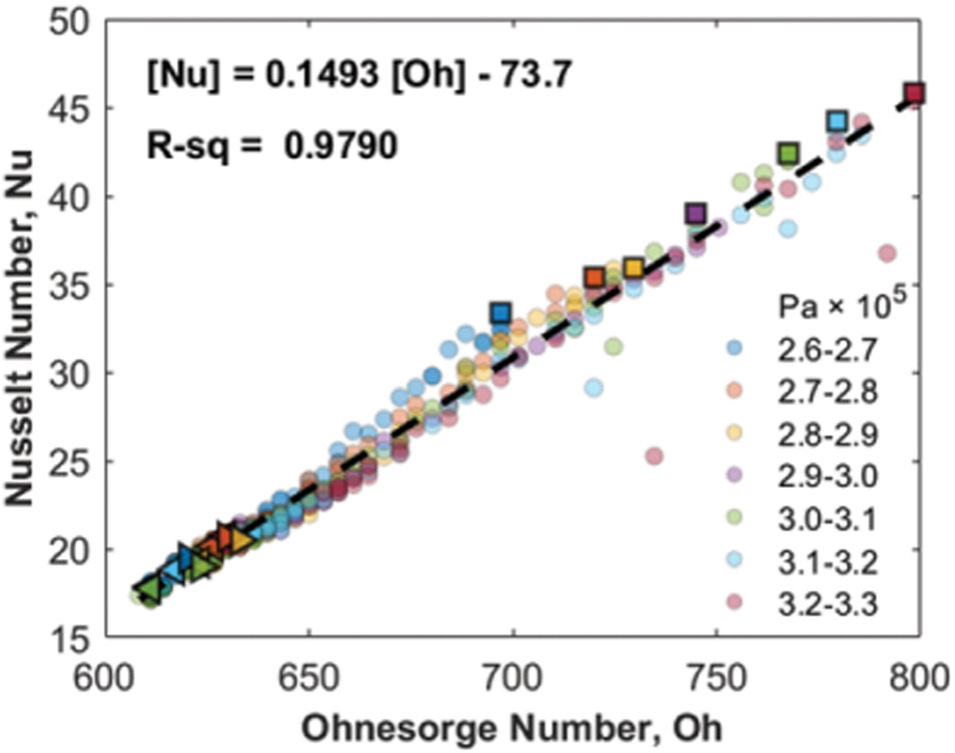
Nusselt number correlation. A universal correlation between the Nusselt number and Ohnesorge number. Special markers share the same definition as in Fig. 9a.

A simple linear correlation is found in Fig. 10 between the Nusselt number and Ohnesorge number for all the 7 peak pressure groups. The Nusselt number increases with increasing Ohnesorge number over the range of the measurements though more experiments are likely needed to determine how long this linear relationship might hold. A higher Ohnesorge number indicates a shorter heat absorption region and a longer heat release region which promotes the heat removal from the hot end. Fig. S.3. demonstrates the length variation of the heat absorption region and heat release region for the nucleation cycle in Fig. 3.

The impact of bubble nucleation on the performance of a wickless heat pipe was studied in microgravity. In the shortest heat pipe tested, nucleation of a second vapor bubble at the heater end boosted the internal pressure of the system due to the generation of extra vapor. The phenomenon was stochastic but based on the peak pressures, the nucleation event cycles were categorized into 7 groups, representing the range of bubbles that could be generated at the heat input applied. A one-dimensional thermal model was used to help quantify the heat transfer processes within the heat pipe, and to extract an average heat transfer coefficient in the region of the pipe associated with heat absorption from the walls. In contrast with the nucleate boiling limits in wick-structured heat pipes, we found the nucleation in the wickless CVB system improved the efficiency of this heat pipe and overcame some of the limitations that arose due to the poor thermal conductivity of the working fluid and the glass walls of the system. During nucleation, the heater wall temperature significantly decreased due to increased evaporation, and the more severe nucleation events cooled the heater wall by 20 degrees or more. The heat transfer coefficient was found to be directly related to the characteristic length of the system, defined as the difference in locations between the heater wall and the point in the heat pipe where the wall temperature coincided with the saturation temperature of the vapor. All the heat transfer data were collapsed into a single, linear *Nu* vs. *Oh* correlation indicating that viscous, surface, and inertial forces dominated the observed behavior.

### Reporting summary

Further information on research design is available in the Nature Research Reporting Summary linked to this article.

## Supplementary information

Temperature Profile

Nucleation Cycle

Nucleation Events in CVB

Supplementary Information

Reporting Summary

## Data Availability

The datasets generated and analyzed during the current study are available from the corresponding author on reasonable request. The raw data is available through NASA’s Physical Science Informatics (PSI) Database under the CVB experiment. [URL: https://www.nasa.gov/PSI].

## Code availability

MATLAB (MathWorks, Inc., Natick, United States) is used to process the data presented in this study. A custom script is available from the corresponding author upon reasonable request.
