# Supplementary material for: The effect of bubble nucleation on the performance of a wickless heat pipe in microgravity
Source: NPJ Microgravity. 2022 Apr 28;8:12. doi: 10.1038/s41526-022-00197-5 (PMC9051110; doi:10.1038/s41526-022-00197-5)
Supplement: Supplementary file 4 — Supplementary Information [file 41526_2022_197_MOESM4_ESM.docx]

Supplementary Information for

**The effect of bubble nucleation on the performance of a wickless heat pipe in microgravity**

Jiaheng Yu

The Howard P. Isermann Department of Chemical and Biological Engineering

Rensselaer Polytechnic Institute

Troy, NY 12180, USA

yuj10@rpi.edu

Anisha Pawar

The Howard P. Isermann Department of Chemical and Biological Engineering

Rensselaer Polytechnic Institute

Troy, NY 12180, USA

pawara@rpi.edu

Joel L. Plawsky

The Howard P. Isermann Department of Chemical and Biological Engineering

Rensselaer Polytechnic Institute

Troy, NY 12180, USA

plawsky@rpi.edu

David F. Chao

NASA Glenn Research Center

Cleveland, OH 44135, USA

david.f.chao@nasa.gov

Corresponding author: Joel L. Plawsky (plawsky@rpi.edu)


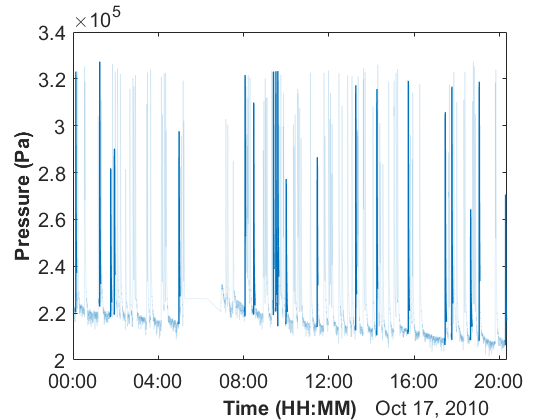


**Fig. S.1.** 20 nucleation cycles selected for the entire analysis. The cycles are highlighted (bold) in the pressure profile.

**Table S.1.** Information of the selected nucleation cycles. 20 cycles were selected for investigation from the total 119 cycles observed through the duration of the experiment. In addition, 7 of them (*stared in the ‘Group’ column) are emphasized in the visualization.

| Group  (Pa × 10^5^) | Peak Pressure  (Pa × 10^5^) | Time interval  (HH:MM:SS) | Number of seconds | Number of data points in the pressure profile, $n_{P}$ | Number of data points in the temperature profile, $n_{T}$ | $n_{T}/n_{P}$ (%) |
| --- | --- | --- | --- | --- | --- | --- |
| 2.6 - 2.7* | 2.6434 | 18:37:58 to 18:43:01 | 303 | 305 | 127 | 41.6 |
| 2.7 - 2.8 | 2.7069 | 20:15:58 to 20:20:01 | 243 | 244 | 102 | 41.8 |
| 2.7 - 2.8* | 2.7724 | 09:58:59 to 10:03:01 | 242 | 243 | 82 | 33.7 |
| 2.8 - 2.9* | 2.8179 | 01:45:00 to 01:49:00 | 240 | 241 | 101 | 41.9 |
| 2.8 - 2.9 | 2.8661 | 11:26:59 to 11:29:01 | 122 | 123 | 42 | 34.1 |
| 2.9 - 3.0* | 2.9020 | 01:56:00 to 01:58:00 | 120 | 121 | 51 | 42.1 |
| 2.9 - 3.0 | 2.9765 | 04:57:59 to 05:01:02 | 183 | 184 | 76 | 41.3 |
| 3.0 - 3.1* | 3.0578 | 17:25:59 to 17:31:02 | 303 | 304 | 127 | 41.8 |
| 3.0 - 3.1 | 3.0992 | 08:26:57 to 08:32:02 | 305 | 306 | 103 | 33.7 |
| 3.1 - 3.2* | 3.1564 | 14:14:00 to 14:17:00 | 180 | 181 | 76 | 42.0 |
| 3.1 - 3.2 | 3.1668 | 17:44:59 to 17:49:02 | 243 | 244 | 102 | 41.8 |
| 3.1 - 3.2 | 3.1730 | 13:14:59 to 13:18:00 | 181 | 182 | 62 | 34.1 |
| 3.1 - 3.2 | 3.1874 | 19:02:30 to 19:04:58 | 148 | 149 | 63 | 42.3 |
| 3.1 - 3.2 | 3.1916 | 15:41:59 to 15:46:52 | 293 | 294 | 123 | 41.8 |
| 3.1 - 3.2 | 3.1936 | 09:34:57 to 09:39:02 | 245 | 246 | 83 | 33.7 |
| 3.2 - 3.3* | 3.2164 | 08:03:00 to 08:05:26 | 146 | 147 | 50 | 34.0 |
| 3.2 - 3.3 | 3.2309 | 00:07:00 to 00:10:00 | 180 | 181 | 76 | 42.0 |
| 3.2 - 3.3 | 3.2316 | 09:22:57 to 09:26:02 | 185 | 186 | 63 | 33.9 |
| 3.2 - 3.3 | 3.2323 | 09:30:00 to 09:34:00 | 240 | 241 | 81 | 33.6 |
| 3.2 - 3.3 | 3.2736 | 01:14:00 to 01:18:00 | 240 | 241 | 101 | 41.9 |

**Table S.2.** Parameters of Antoine equation. Pressure, $P$, is in mmHg; saturation vapor temperature, $T_{v}$, is in K.

|  | A | B | C |
| --- | --- | --- | --- |
| Pentane | 7.00877 | 1134.15 | 238.678 |


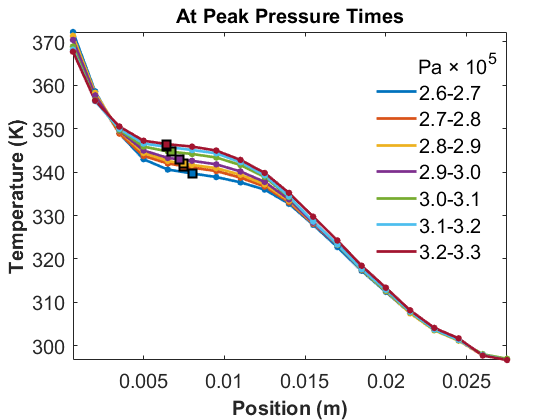


**Fig. S.2.** Temperature profiles at the peak pressure times for the 7 groups. The square dots represent the saturation vapor temperatures.

**Table. S.3.** Physical properties of liquid pentane in the calculation of the Ohnesorge number (Eq. (4)) and Nusselt number (Eq. (5)).

| Physical Property  (T = 335 K) | Viscosity  cP  $\mu$ | Density  kg/m^3^  $\rho$ | Surface Tension  N/m  $\sigma_{l}$ | Thermal Conductivity  W/(m K)  $k$ |
| --- | --- | --- | --- | --- |
| Value | 0.16153 | 582.57 | 0.011523 | 0.098066 |

| 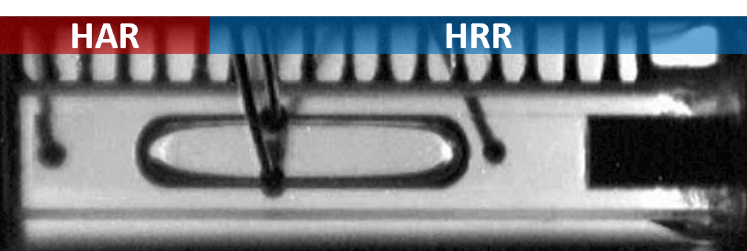 | 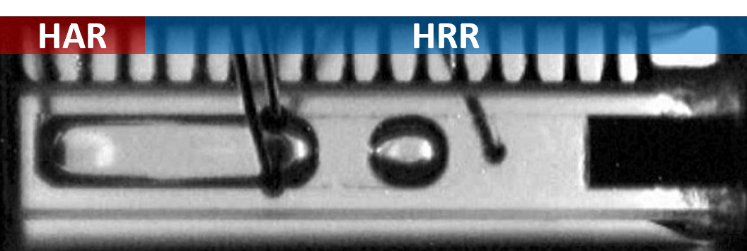 |
| --- | --- |
| 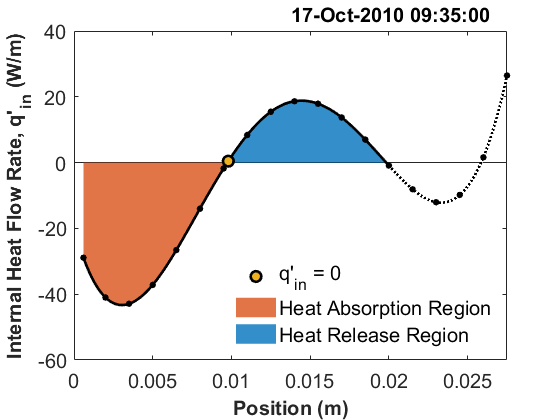 | 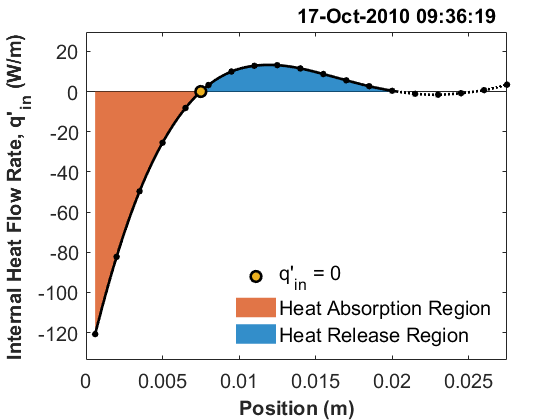 |
| (a) | (b) |
| 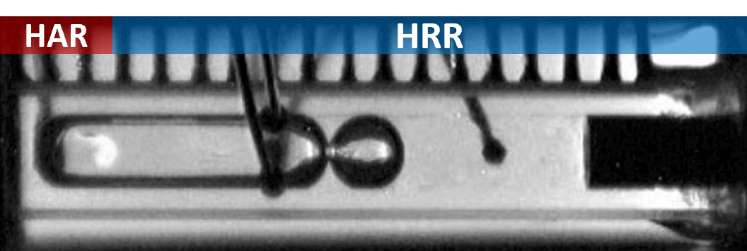 | 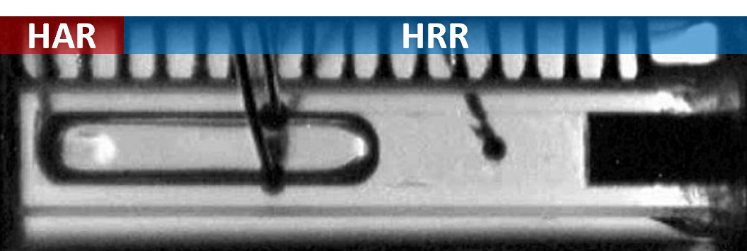 |
| 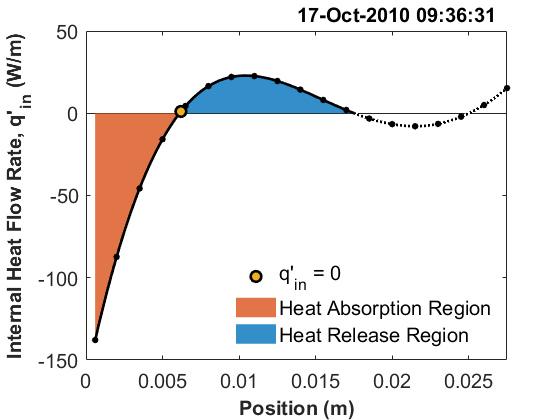 | 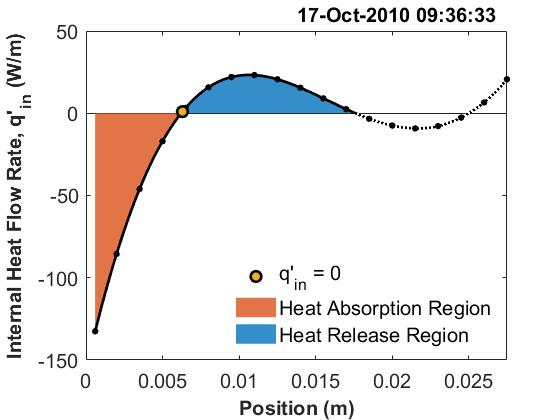 |
| (c) | (d) |

| 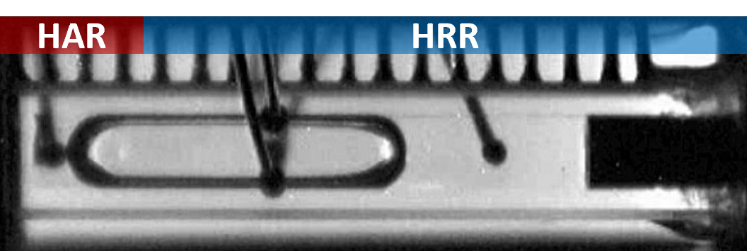 | 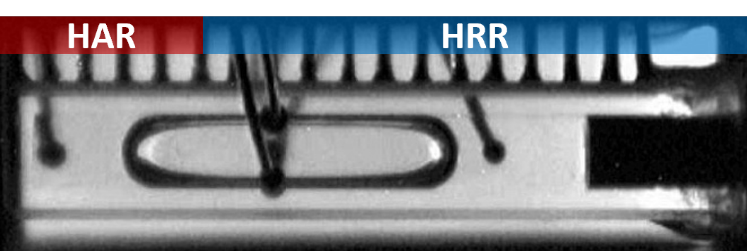 |
| --- | --- |
| 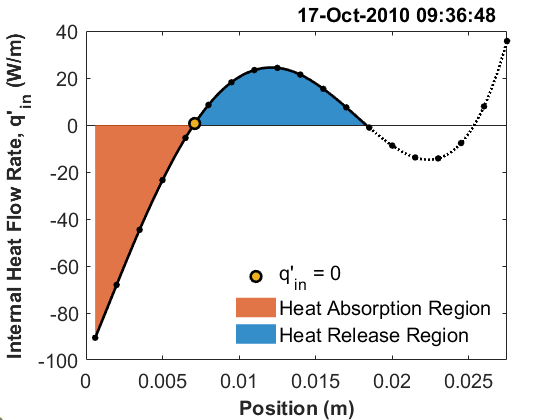 | 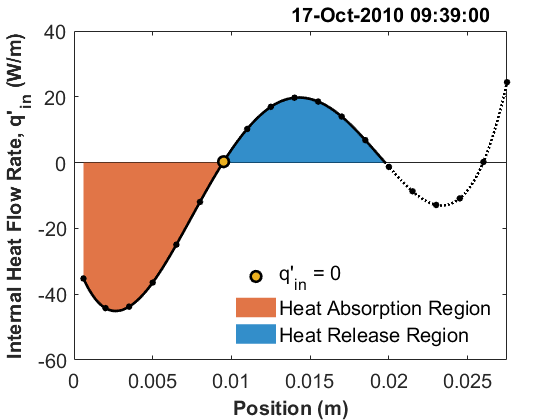 |
| (e) | (f) |

**Fig. S.3.** Length of the heat absorption region (HAR) and heat release region (HRR). The internal heat flow rate, $q’_{in}$, is used to determine the length of the two regions. The $q’_{in}$ profiles are shown for the six stages of the nucleation cycle in Fig. 3.


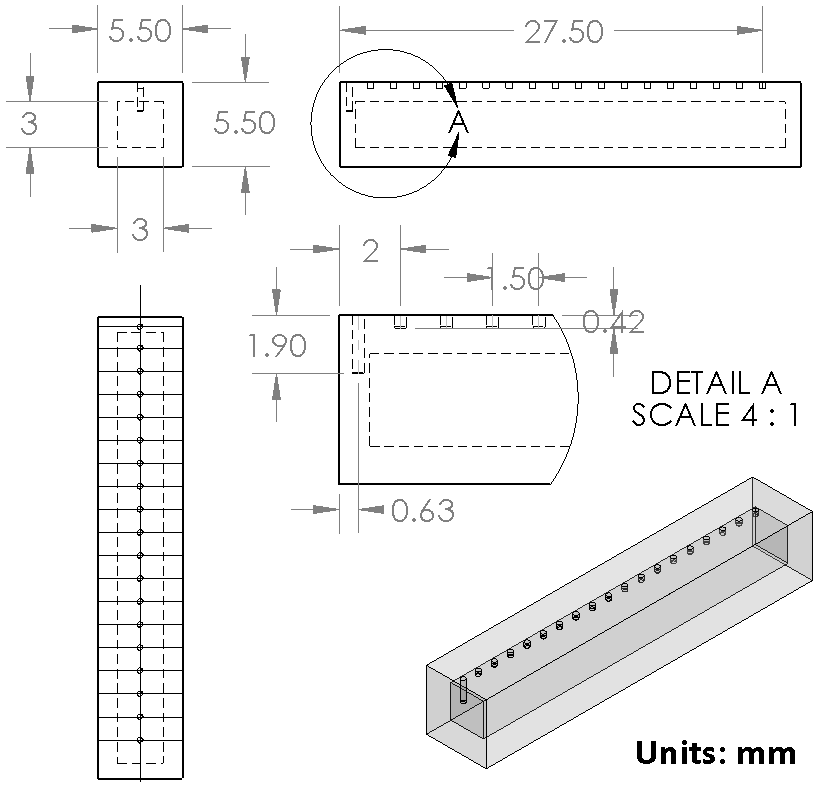


**Fig. S.4.** Drilled wells on the cuvette surface for thermocouple placement. The diagram shows a section view near the heater end. The first deep well located at the first thermocouple, TC#01, measures the heater wall temperature. Other shallow wells contained the thermocouples that measured the temperature profile along the main axis.
